# Rehabilitation of gingival architecture by a conservative method: An innovative approach

**DOI:** 10.4103/0972-0707.45253

**Published:** 2008

**Authors:** Abhishek Parolia, Manuel S Thomas, M Kundabala, Neeta Shetty, Suman Gautam, Senthil Kumar

**Affiliations:** Department of Conservative Dentistry and Endodontics, Manipal College of Dental Sciences, Mangalore, Karnataka, India

**Keywords:** Ceramic tints, composite resin, esthetic rehabilitation, gingival recession

## Abstract

Periodontal attachment loss in the maxillary anterior region can often lead to esthetic and functional clinical problems. Lifelong motivation is essential to the supportive therapy for these patients, and the maintenance of good esthetics, combined with conducive to maintaining long term dental and professional health. This paper aims to demonstrate an innovative treatment option for dealing with aesthetic challenges posed by a number of patients who have undergone initial cause related therapy for aggressive periodontitis.

## INTRODUCTION

Awareness about various dental treatment modalities has increased among the patients now, as compared to what it was a decade ago.

Oral esthetic and functional rehabilitation can be achieved by restoring teeth and soft tissue defects to an ideal natural form. Ideal gingival esthetics may not be achievable in a compromised case. Gingival replacement prosthesis has historically been used to replace lost tissue, when other methods such as surgery or regenerative procedures were considered unpredictable or impossible. It is very important to take care of function, comfort, phonetics, longevity and ease of maintenance, with preferably less cost. Materials used for replacing lost tissue architecture include pink auto cure and heat cure acrylics, porcelains, composite resins, as well as silicon based soft materials.[1] Gingival defect may be treated with high cost surgery, following which healing may take a longer time, with unpredictable results, therefore making the choice unpopular.

The following case reports describe a new technique for replacing the gingival tissue by an easier and quicker method, which is more economical for the patient. In all these cases, there was gingival recession following active periodontal treatment and the resolution of inflammation in periodontitis; the periodontal tissue was healthy, but with a reduced connective tissue attachment and alveolar bone height.[2]

## CASE REPORTS

### Case 1

A 25-year-old female patient presented to the Department of Periodontics, Manipal College of Dental Sciences, Mangalore, in August 2004, with a primary complaint of mobility of right lateral incisor (12) and spacing between right lateral and central incisor. On examination, the right lateral incisor showed grade II mobility, with probing depth of 7.0 mm. After thorough oral prophylaxis, flap surgery was done, resulting in gingival shrinkage and exposed roots. Postoperative checkup after three months showed that the disease was under control, with good oral hygiene maintenance, but the patient was still concerned about the spacing and mobility. As the prognosis for further periodontal treatment was poor, the patient was advised to go for extraction of the tooth and replacement with a removable or fixed prosthesis. Since the patient was not interested in extraction and wanted to retain the tooth, she was referred to the Department of Conservative Dentistry for further esthetic management [Figure 1A-Pre-treatment]. A treatment plan was established involving the following steps:

1. The topography of the soft tissue defect was evaluated.
2. The color of the soft tissue was determined, to achieve the acceptable esthetics with tissue colored porcelain tints.
3. Shade and contour of gingiva and teeth were confirmed by curing the composite resin material over the lost tissue portion, without application of etchant and bonding agent.
4. Treatment was also planned to close the diastema and restore the exposed root surface using composite resin.

**Figure 1A:**
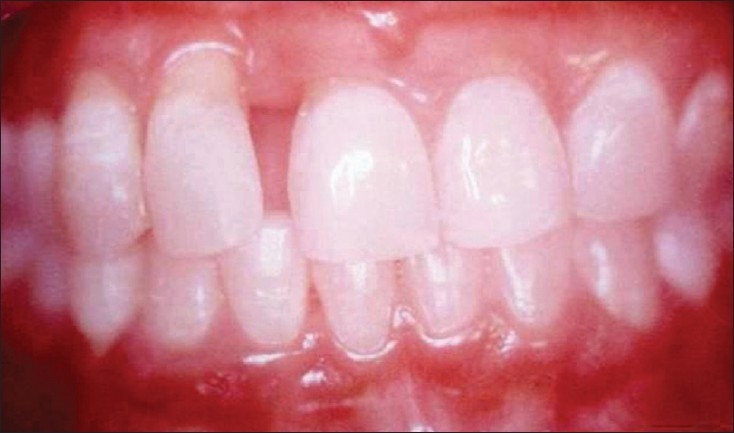
Pre-treatment

### Procedure

The teeth were isolated, acid etched with 35% phosphoric acid (3M ESPE Dental Products USA) for 15 seconds and blot dried. Bonding agent (Adper Single Bond 2 3M ESPE Dental Products USA) was applied and light cured, according to the manufacturer's instructions. Incremental composite resin (3M ESPE Dental Products USA) build up was done for the coronal tooth structure, to close the diastema. Ceramic tint powder (Number 703 and 704, Vita GmbH and Co.KG Germany) was taken in a well, to match the shade of the gingiva. One to two drops of dentin bonding agent was added to dissolve the ceramic tint in it. Composite resin material of selected shade was added to the mixture, till a thick paste consistency was achieved. The gingiva was built up to the proper contour, without any overhang of material. The material was then light cured. Finishing polishing was done using composite finishing and polishing system (Enhance Dentsply Caulk Dentsply International Inc. Milford). A fiber composite splint was placed for two months, from canine to canine, for stabilizing the teeth to reduce the mobility [Figure 1B-Post-treatment].

**Figure 1B:**
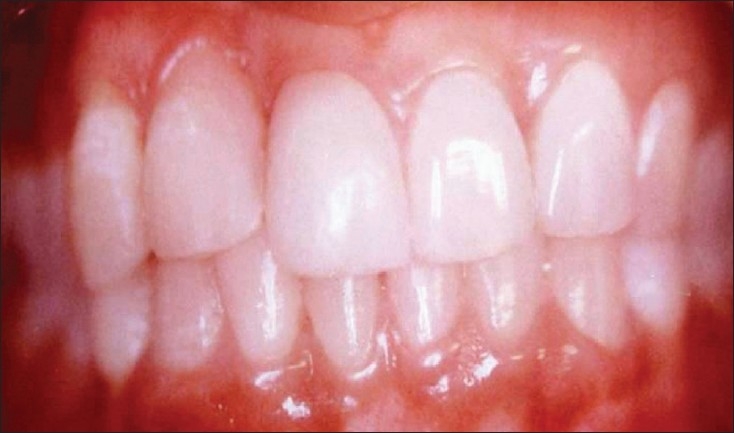
Post-treatment

### Case 2

A 25-year-old female patient presented to Department of Conservative Dentistry and Endodontics, with a complaint of gap between her front teeth. The patient expressed her concern about her unesthetic appearance, because of the space between the central incisors. On examination of 11 and 21, the following were noted [Figure 2A-Pre-treatment].

- Diastema of about 3.0 mm incisally and 4.0 mm gingivally.
- Gingival recession and root exposure on the labial surface of both 11 and 21.
- Abrasion of the labial surface of 11 and 21.

On periodontal examination, there was no bleeding on probing and absence of periodontal pockets.

**Figure 2A:**
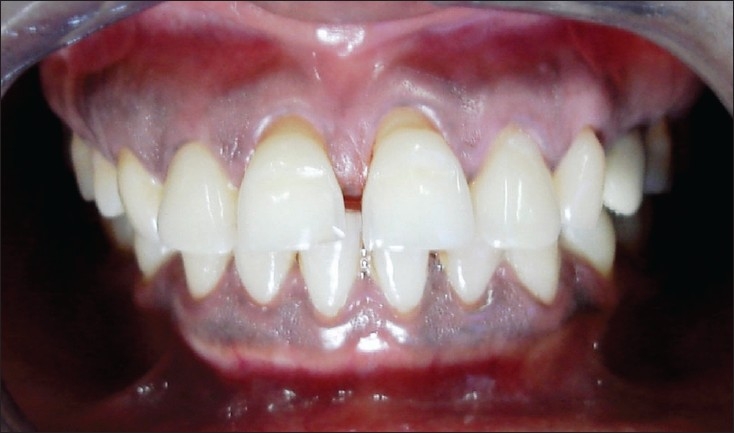
Pre-treatment

### Treatment planning

Treatment was planned to close the diastema and to restore the soft tissue defect. Teeth and soft tissue defects were treated in a similar fashion, as described in Case 1. The ceramic tints chosen were Number 704 and 708, Vita GmbH and Co.KG Germany, in this case. Composite resin was blended with the root surfaces and care was taken not to impinge the soft tissue [Figure 2B-Post-treatment]. The contacts were checked using dental floss. Postoperative instructions were given to the patient, who was recalled after one, three and six months for follow up. The original contour and shade were maintained with good oral hygiene.

**Figure 2B:**
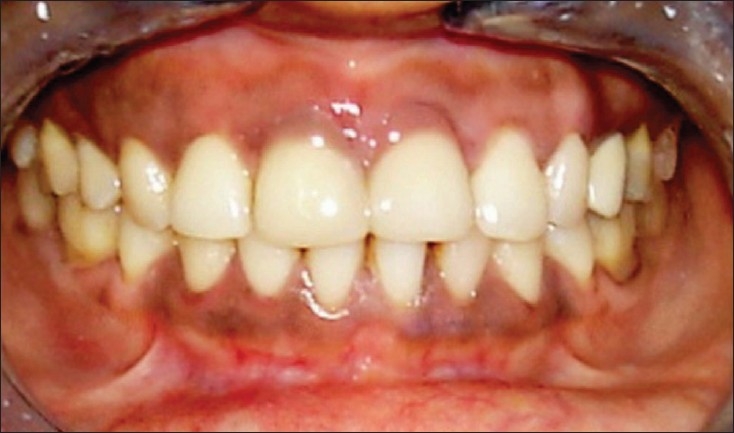
Post-treatment

### Case 3

A female patient, 24 years of age, had come to the Department of Conservative dentistry and Endodontics, with a primary complaint of spacing between her upper anterior teeth. Past dental history suggested aggressive periodontitis, for which flap surgery was performed in the Department of Periodontics six months back. The patient had been recalled after one, three and six months for review. After healing, gingival shrinkage and root exposure was noticed. The lower anterior teeth were splinted with composite fiber splint, as they were mobile. Once the disease was seen to be under control and the prognosis found to be satisfactory, the patient was referred for esthetic rehabilitation.

Dental examination revealed diastema between her upper anterior teeth.

- Triangular space of approximately 2 mm was seen between 11 and 21
- Diastema of 2 mm was seen between 11 and 12
- Diastema of 1 mm was seen between 21 and 22

The spacing was created due to pathological migration and papillary recession.

Gingival recession was present.

- Recession of 1.5 mm from the cemento-dentinal junction on the labial aspect of 12
- Recession of 2.5 mm from the cemento-dentinal junction on the labial aspect of 11
- Recession of 1.0 mm from the cemento-dentinal junction on the labial aspect of 21

The upper central incisors were slightly extruded because of the previous periodontal problem. No significant mobility was seen in the upper anterior teeth. The gingiva was healthy, with no bleeding and periodontal pockets on probing [Figure 3A-Pre-treatment].

**Figure 3A:**
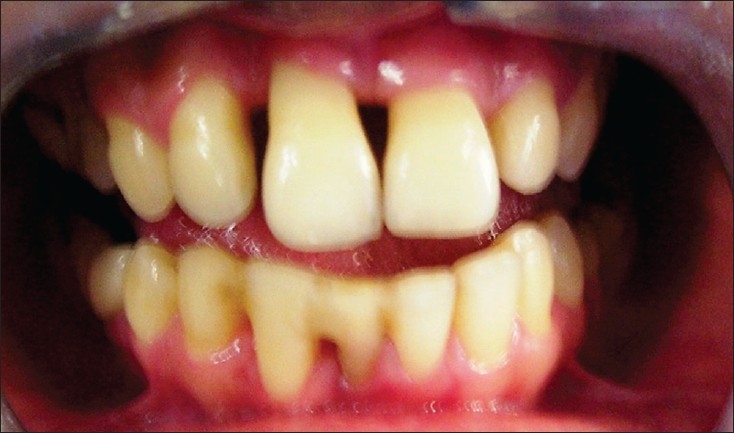
Pre-treatment

The treatment plan consisted of creating acceptable esthetics by recontouring the incisal edges, closing the diastema using composite resin and reproducing the receded gingival architecture using pink composite resin. The resin was prepared by adding the ceramic tint powder (Number 703 and 704, Vita GmbH and Co.KG Germany), to match the shade of gingiva, to one to two drops of dentin bonding agent and mixing it with the composite resin material [Figure 3B-Post-treatment].

**Figure 3B:**
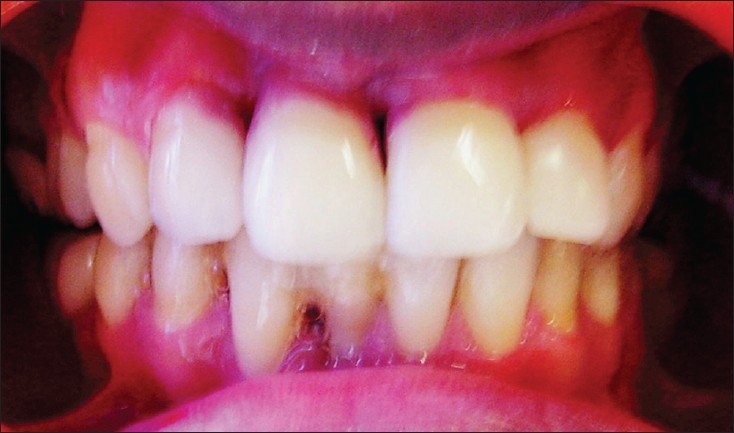
Post-treatment

### Case 4

An eighteen-year-old female patient came to the Department of Conservative Dentistry with a primary complaint of long front teeth. The patient had a history of undergoing flap surgery of her gums four months back. On examination, there was presence of gingival recession, along with pathological migration (12, 11 and 21, 22) of anterior teeth, leading to compromise in the esthetic appeal of the patient [Figure 4A-Pre-treatment]. Since the patient was highly concerned about her smile, a treatment plan was formulated to close the diastema and the soft tissue defect with composite restoration using ceramic tint (Number 704, Vita GmbH and Co.KG Germany) in the cervical and the root surface area, to mimic gingival contour. Composite resin was blended with root surfaces and care was taken not to impinge the soft tissue [Figure 4B-Post-treament]. The contacts were checked using dental floss. Postoperative instructions were given to the patient. On the follow-up after six months, the original contour and shade were maintained with good oral hygiene.

**Figure 4A:**
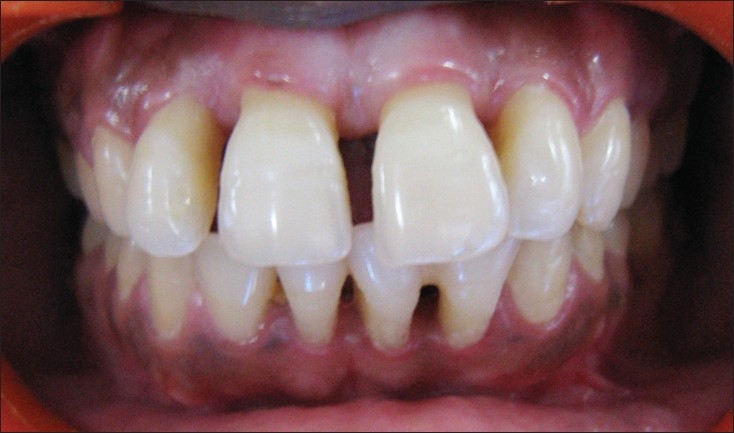
Pre-treatment

**Figure 4B:**
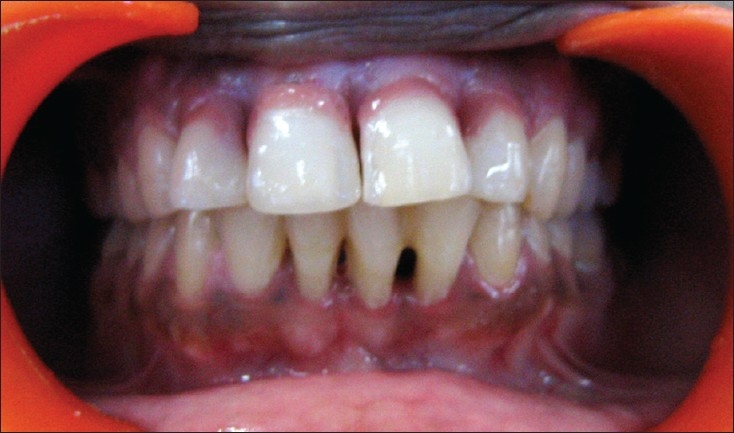
Post-treatment

## DISCUSSION

Some of the young patients who have successfully completed treatment for aggressive periodontitis may have implications in terms of long term restorative maintenance. Restorations need to be carefully reviewed, maintained and replaced throughout the patient's life and the lifespan of some restorative options need to be carefully evaluated.

There are various treatment options to meet these challenges. These include:[3]

1. Treatment with restorative camouflage
2. Treatment with extraction and immediate fixed replacement
3. Treatment with fixed appliance orthodontics
4. Treatment with gingival prosthesis
5. Treatment with ceramic crowns
6. Treatment with gingival resin from Ivoclar, Tragis to restore the gingiva

The innovative technique used here is a quicker (single sitting) and simpler one. No elaborate laboratory procedures are involved. The porcelain stains used in this technique, to get the shade of gingiva, readily dissolved in the dentin bonding agent, which shows that they must be chemically compatible with each other. The stains must have acted like mega fillers in the composite resin. They merged very well and this could be estimated by the high polishability of the restoration.

The advantages of this technique are as follows:

- There is no composite resin impingement on to the soft tissue.
- Composite resin blends well with the root surface.
- Floss can be easily moved on and off the contact to maintain the oral hygiene
- The ceramic tints which are used in this technique help to get the exact shade of lost gingival tissue, that may be difficult to achieve with composite stains.
- Though Ivoclar, Tragis resin material is available, the exact shade of gingiva may be achieved with this technique, at less cost.

Fiber composite splint was used in Case 1, to reduce the mobility of the tooth. Since we closed the diastema in this case, we could apply the composite splints on the palatal surface, without compromising esthetic needs, thus improving their function.

In all the cases, shade selection was done by curing the composite resin material directly on the tooth structure, without acid etching and application of the bonding agent. Once the shade and contour were confirmed, composite material was nicked off the tooth structure. The procedure was repeated till the proper shade and contour were achieved, without harming the tooth structure. Acid etchant and dentin bonding agent were applied prior to building up the tooth structure with composite resin, only for the final restoration. Further studies have to be done to find out the chemistry of the mixture (bonding agent with ceramic tints) and long term evaluation of the cases, to check the outcome of the treatment.

## CONCLUSION

For patients with periodontally compromised teeth, not willing for extraction, this technique is an option which is quicker, simple, more economical and facilitating conservation of the remaining natural tissues. Thorough clinical examination should be carried out and the patient should be fully involved in the decision making, during the treatment planning.
